# Serum Anti-BRAT1 is a Common Molecular Biomarker for Gastrointestinal Cancers and Atherosclerosis

**DOI:** 10.3389/fonc.2022.870086

**Published:** 2022-05-17

**Authors:** Liubing Hu, Jiyue Liu, Hideaki Shimada, Masaaki Ito, Kazuo Sugimoto, Takaki Hiwasa, Qinghua Zhou, Jianshuang Li, Si Shen, Hao Wang

**Affiliations:** ^1^ Stroke Center, The First Affiliated Hospital, Jinan University, Guangzhou, China; ^2^ The Biomedical Translational Research Institute, Faculty of Medical Science, Jinan University, Guangzhou, China; ^3^ College of Life Science and Technology, Jinan University, Guangzhou, China; ^4^ Department of Anesthesiology, The First Affiliated Hospital, Jinan University, Guangzhou, China; ^5^ Department of Gastroenterological Surgery and Clinical Oncology, Toho University Graduate School of Medicine, Tokyo, Japan; ^6^ Department of Biochemistry and Genetics, Graduate School of Medicine, Chiba University, Chiba, Japan; ^7^ Department of Neurology, Dongzhimen Hospital, Beijing University of Chinese Medicine, Beijing, China; ^8^ Department of Radiology, Medical Imaging Center, The First Affiliated Hospital, Jinan University, Guangzhou, China

**Keywords:** antibody biomarker, atherosclerosis, BRAT1, gastrointestinal cancer, liquid biopsy

## Abstract

Atherosclerosis (AS) and cancers are major global causes of mortality and morbidity. They also share common modifiable pathogenesis risk factors. As the same strategies used to predict AS could also detect certain cancers, we sought novel serum antibody biomarkers of cancers in atherosclerotic sera sampled by liquid biopsy. Using serological antigen identification by cDNA expression cloning (SEREX) and western blot, we screened and detected the antigens BRCA1-Associated ATM Activator 1 (BRAT1) and WD Repeat Domain 1 (WDR1) in the sera of patients with transient ischemic attacks (TIA). Amplified luminescence proximity homogeneous assay-linked immunosorbent assay (AlphaLISA) established the upregulation of serum BRAT1 antibody (BRAT1-Abs) and WDR1 antibody (WDR1-Abs) in patients with AS-related diseases compared with healthy subjects. ROC and Spearman’s correlation analyses showed that BRAT1-Abs and WDR1-Abs could detect AS-related diseases. Thus, serum BRAT1-Abs and WDR1-Abs are potential AS biomarkers. We used online databases and AlphaLISA detection to compare relative antigen and serum antibody expression and found high BRAT1 and BRAT1-Abs expression in patients with GI cancers. Significant increases (> 0.6) in the AUC for BRAT1-Ab vs. esophageal squamous cell carcinoma (ESCC), gastric cancer, and colorectal cancer suggested that BRAT1-Ab exhibited better predictive potential for GI cancers than WDR1-Ab. There was no significant difference in overall survival (OS) between BRAT1-Ab groups (*P* = 0.12). Nevertheless, a log-rank test disclosed that the highest serum BRAT1-Ab levels were associated with poor ESCC prognosis at 5–60 weeks post-surgery. We validated the foregoing conclusions by comparing serum BRAT1-Ab and WDR1-Ab levels based on the clinicopathological characteristics of the patients with ESCC. Multiple statistical approaches established a correlation between serum BRAT1-Ab levels and platelet counts. BRAT1-Ab upregulation may enable early detection of AS and GI cancers and facilitate the delay of disease progression. Thus, BRAT1-Ab is a potential antibody biomarker for the diagnosis of AS and GI cancers and strongly supports the routine clinical application of liquid biopsy in chronic disease detection and diagnosis.

## Introduction

Atherosclerosis (AS) and cancers account for the majority of global morbidity and mortality (1). AS is a major cause of coronary artery diseases such as acute myocardial infarction (AMI) and ischemic cerebrovascular diseases such as transient ischemic attacks (TIA), cerebral infarction (CI), and peripheral vascular disease. Its major risk factors include tobacco smoking, obesity, diabetes mellitus (DM), hypertension, and hypercholesterolemia (2, 3). Gastrointestinal cancers [esophageal (EC), gastric (GC), liver (LC), colorectal (CRC), and pancreatic (PC)] account for 26% of all cancer incidences and 35% of all cancer-related deaths worldwide (4, 5). Therefore, biomarkers that accurately predict AS-related diseases and GI cancers, provide early diagnosis, improve prophylactic and therapeutic strategies, and reduce disease burdens are urgently needed (6).

Several studies have suggested that AS may be correlated with GI cancers. AS might affect GI cancer progression and vice-versa. They share several common risk factors including tobacco smoking, obesity, and DM (7). Certain common molecular pathways, metabolic disorders, hereditary alterations, and lifestyle practices are also correlated with AS and cancer development (6). Some anti-atherosclerotic agents also have efficacy against GI cancers (8). These drugs include metformin (9), aspirin (10), and statins (11). Another study identified the human gut microbiome as a putative common therapeutic target for AS and cancer (8). It has been extensively demonstrated that patients with AS are at risk of certain cancers. A multi-ethnic study on AS reported that coronary artery calcification is correlated with elevated risks of lung cancer and CRC (12). The prevalence of colorectal adenoma was relatively higher in patients with significant coronary artery disease or low-grade coronary AS (13). Increased urogenital or gastrointestinal bleeding was associated with new cancer diagnoses in subjects with AS undergoing antithrombotic therapy (14). Another study explored the application of strategies that could simultaneous prevent atherosclerotic vascular disease and certain cancers (6). The results of the foregoing studies suggest that risk markers related to AS diseases may also predict cancer occurrence and prognosis.

Liquid biopsy is used to examine biomarkers in body fluids (15). Compared with tissue biopsy and imaging, liquid biopsy is minimally invasive, facilitates sample collection, tracks the entire disease course, and is cost-effective. Liquid biopsy analyses are performed on different body fluids to sample circulating tumor cells (CTCs), circulating tumor DNA (ctDNA), proteins such as serum autoantibodies, cell-free RNAs (mRNAs and microRNAs), metabolites, and so on (16).

AS is a chronic inflammatory disease associated with autoimmunity. Hence, the plaques it deposits contain autoantibodies (17, 18). Serological antigen identification by cDNA expression cloning (SEREX) is a liquid biopsy method and an effective technique for screening antigen and antibody markers (19, 20). SEREX screens antigens related to tumors and autoimmune diseases including GI cancers [EC (21, 22), GC (23), LC (24), and CRC (25) and PC (26)], multiple sclerosis (MS) (27), systemic sclerosis (SSc) (28), systemic lupus erythematosus (SLE) (29), rheumatoid arthritis (RA) (30), and others. In earlier studies, we successfully applied SEREX to AS-related diseases and identified antibodies against CPSF2, DIDO1, FOXJ2, MMP1, CBX1, CBX5, and LAMP1 in TIA and CI (31–33). We also identified antibodies against TUBB2C in the sera of DM patients (34). Our laboratory previously found that antibodies against LRPAP1 (35) and ASXL2 (36) were upregulated in patients with AS-related diseases and GI cancers. Hence, LRPAP1-Ab and ASXL2-Ab may be biomarkers common to all these disorders. However, we hope to use AS markers to predict GI cancer occurrence and prognosis and explore other markers common to both types of conditions.

In the present study, SEREX revealed that BRAT1-Ab and WDR1-Ab are AS disease biomarkers. Subsequent exploration of a cancer database showed that BRAT1 was upregulated in GI cancers. Serological verification disclosed that BRAT1-Ab was also a GI cancer biomarker. These discoveries suggest that BRAT1 is a common biomarker of AS diseases and GI cancers and these disorders are correlated. Moreover, the foregoing results indicate that liquid biopsy could achieve early AS and cancer diagnosis in clinical practice and help forecast the outcomes of these conditions.

## Materials and Methods

### Sera from Patients and Healthy Donors

Nineteen TIA patients from Chiba Rosai Hospital were randomly selected for SEREX immunoscreening. To compare antibody levels, sera were acquired from 92 patients with TIA, 464 patients with acute cerebral infarction (aCI), and 65 patients with old cerebral infarction (oCI) at Chiba Prefectural Sawara, Chiba Rosai, and Chiba Aoba Municipal Hospitals. Sera were also acquired from 128 DM and 128 AMI patients at Chiba and Kyoto University Hospitals, respectively. Sera were acquired from 192 patients with ESCC, 96 patients with GC, and 191 patients with CRC at Toho University Omori Hospital. All subjects with ESCC were followed up until January 2020 or their death. Sera of healthy donors (HDs) were provided by Port Square Kashiwado Clinic, Chiba Prefectural Sawara Hospital, and Toho University Omori Hospital. These subjects had no aberrant cranial resonance imaging. The sera were centrifuged at 3,000 × *g* for 10 min and the supernatants were stored at -80°C until use. Sample freeze-thaw was avoided.

### Immunological Screening by SEREX

An improved version of the aforementioned method was used to screen clones that were immunoreactive to the sera of patients with TIA (31). A human aortic endothelial cell cDNA expression library (Uni-ZAP XR Premade Library, Stratagene, La Jolla, CA, USA) was transfected into *Escherichia coli* (*E. coli*) XL1−Blue MRF′ (Stratagene). The resident cDNA clones were transferred onto nitrocellulose (NC) membranes pretreated with 10 mM isopropyl-β-D-thiogalactoside (IPTG; Wako Pure Chemicals Industries Ltd., Osaka, Japan) for 30 min. Membranes with bacterial proteins were washed thrice with TBS-T (20 mM Tris-HCl (pH 7.5), 0.15 M NaCl, and 0.05% (w/v) Tween 20). Then the membranes were incubated for 1 h in 1% (w/v) protease-free bovine serum albumin (BSA; Nacalai Tesque, Inc., Kyoto, Japan) in TBS-T to block nonspecific binding. The membranes were then incubated overnight with diluted sera (1:2,000) from the TIA patients. The membranes were washed thrice in TBS−T and incubated for 1 h in alkaline phosphatase−conjugated goat anti−human IgG (1:5,000; Jackson Immuno Research Laboratories, West Grove, PA, USA). Positive responses were identified by cultivating the membranes in a color development solution (100 mM Tris-HCl (pH 9.5), 100 mM NaCl, and 5 mM MgCl_2_) containing 0.15 mg/mL 5-bromo-4-chloro-3-indolyl phosphate (Wako Pure Chemicals Industries Ltd.) and 0.3 mg/mL nitroblue tetrazolium (Wako Pure Chemicals Industries Ltd.). Cloning was performed twice on the positives until monoclonality.

### Sequence Analysis of Identified Antigens

ExAssist helper phage (Stratagene) and *in vitro* excision were used to convert the monoclonalized phage cDNA clones into pBluescript phagemids. Plasmid DNA was extracted from the *E. coli* SOLR strains transformed by the phagemids. The infused cDNAs were sequenced for homology using the public database provided by the National Center for Biotechnology Information (http://www.ncbi.nlm.nih.gov/Blast.cgi/).

### Expression Vector Construction

To construct the glutathione-*S*-transferase (GST)-fused protein expression plasmids, the cDNA sequences were recombined into the pGEX-4T vector (GE Healthcare Life Sciences, Pittsburgh, PA, USA) as previously described (32, 35, 37). The pBluescript plasmids associated with the cDNA inserts were digested with the restriction endonucleases *Eco*RI and *Xho*I and detached by agarose gel electrophoresis. GenElute Minus EtBr spin columns (Sigma-Aldrich Corp., St. Louis, MO, USA) were used to isolate the cDNA fragments which were ligated in frame to *Eco*RI- and *Xho*I-digested pGEX-4T-3 linearized vectors with ligation convenience kits (Nippon Gene, Toyama, Japan). The ligation mixtures were used to transform ECOS-competent *E. coli* BL-21 cells (Nippon Gene). Successful recombination was confirmed by DNA sequencing and protein expression analysis.

### Recombinant Candidate Protein Purification

*Escherichia coli* BL-21 cells transformed with the pGEX-4T clone were cultured in 200 mL Luria-Bertani (LB) broth and treated with 1 mM IPTG for 3 h. The cells were collected in bacterial solution and lysed by sonication in BugBuster Master Mix (Novagen, San Diego, CA, USA). The lysates were then centrifuged at 13,000 × *g* and 4°C for 10 min. GST-tagged BRAT1 and GST-tagged WDR1 proteins were purified by glutathione-Sepharose column chromatography (GE Healthcare Life Sciences) and dialyzed as previously described (32, 36, 38).

### Western Blotting

Purified GST, GST-BRAT1, and GST-WDR1 proteins (0.3 μg) were separated by SDS-PAGE. After transfer and blocking, anti-GST (Rockland, Gilbertsville, PA, USA) or serum (1:5,000) from patients with TIA (#297) was used as a source of primary antibodies. The proteins were then incubated with HRP-conjugated secondary antibody (donkey anti-goat or anti-human IgG; Santa Cruz Biotechnology, Dallas, TX, USA) and detected as previously described (32, 39).

### AlphaLISA of Antibody Biomarkers and Conventional Serum Marker Measurement

AlphaLISA was used to quantify the serum antibodies against the purified proteins. The α-luminescent photon counts represent the serum antibody levels (35, 40). The AlphaLISA assay was performed in 384-well microtiter plates (white opaque OptiPlate; PerkinElmer, Waltham, MA, USA). Each well contained 2.5 μL serum (1:100 dilution) and 2.5 μL GST or GST fusion protein (10 μg/mL) in AlphaLISA buffer (25 mM HEPES [pH 7.4], 0.1% (w/v) casein, 0.5% (w/v) Triton X-100, 1 mg/mL Dextran-500, and 0.05% (w/v) Proclin-300). The mixture was then incubated at 25°C for 8 h. Then 2.5 μL of 40 μg/mL anti-human IgG-conjugated acceptor beads and 2.5 μL of 40 μg/mL glutathione-conjugated donor beads were added and the mixture was incubated in the dark at 25°C for 7–21 d. Chemical emission was measured in an EnSpire Alpha microplate reader (PerkinElmer) as previously described. The reactions were calculated by subtracting the Alpha counts for the GST control from those for the GST fusion proteins.

The levels of serum squamous cell carcinoma antigen (SCC-Ag) (41) and p53 antibody (p53-Abs) (42) were evaluated as previously described. The serum SCC-Ag and p53-Abs cutoff values were 1.5 ng/mL and 1.3 IU/mL, respectively.

### BRAT1 Expression Analysis

Tumor Immune Estimation Resource (TIMER2.0, http://timer.cistrome.org/) estimates the immune invasion levels of numerous cancers. The “Gene_DE” module permits users to compare gene expression levels between normal tissues and those of tumors associated with all 32 types of cancer listed in The Cancer Genome Atlas (TCGA) cohort (43, 44). C7orf27 (BRAT1) or WDR1 were inserted into the “Gene_DE” module of TIMER2.0 Web to analyze the differences in BRAT1 expression between normal tissues and the tumors listed in the TCGA cohort.

Gene Expression Profiling Interactive Analysis (GEPIA, http://gepia2.cancer-pku.cn/) analyzes gene expression in tumor and normal samples from the TCGA and Genotype-Tissue Expression (GTEx) databases (45, 46). GEPIA was used to analyze BRAT1 expression in various tumors in the TCGA cohort. Matching normal TCGA data served as the control.

The TNMplot (http://www.tnmplot.com) database contains 56,938 samples of normal, tumor, and metastatic tissues from gene chip studies, TCGA, Therapeutically Applicable Research to Generate Effective Treatments (TARGET), and GTEx (47). TNMplot was used to determine BRAT1 expression levels in different tumor tissues and compare them against the BRAT1 expression profiles of normal tissues.

UALCAN (http://ualcan.path.uab.edu) is an interactive web portal containing TCGA clinical data for 31 cancer types and RNA-seq. UALCAN has been used for in-depth TCGA gene expression data analysis (48). UALCAN can also analyze protein expression using data from the Clinical Proteomic Tumor Analysis Consortium (CPTAC, http://ualcan.path.uab.edu/analysis-prot.html). Here, UALCAN was used to analyze protein expression.

### Statistical Analyses

Student’s *t*-test and Mann-Whitney *U* test were used to analyze differences between group pairs. Correlations among Alpha values and clinical data were calculated by multivariate logistic regression and Spearman’s correlation analyses. Differences in the distributions of two variables were calculated by Fisher’s exact test. The predictive values of the disease markers were assessed by ROC analysis. The antibody level cutoff values were set to maximize the sensitivity and specificity sums. Survival curves were plotted by the Kaplan-Meier method. Comparisons were made *via* the log-rank test. The Cox proportional hazards model was used to evaluate significant predictors. All tests were two-tailed. *P* < 0.05 was considered statistically significant. GraphPad Prism 6 (GraphPad Software, La Jolla, CA, USA) was used to perform all statistical analyses.

## Results

### Autoantibodies Against Purified BRAT1 and WDR1 Proteins are Present in Sera of Patients with TIA

**Figure 1:**
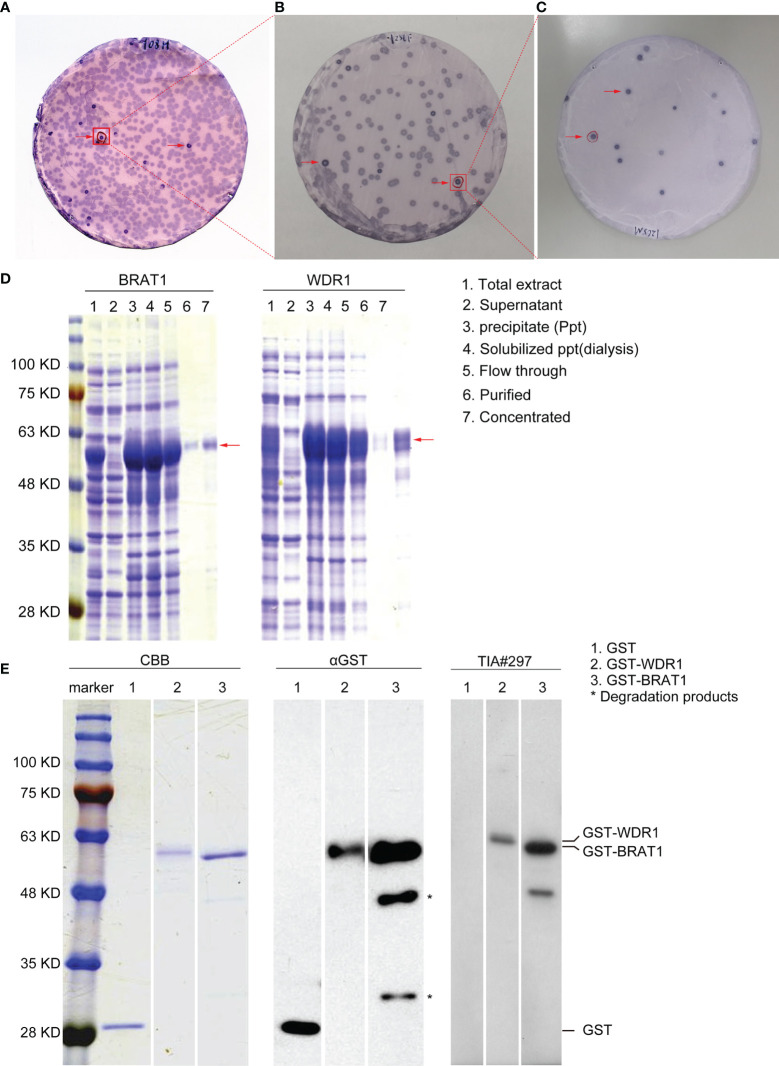
Recognition and identification of BRAT1 and WDR1 using serum of TIA patients. **(A)** Recombinant expression cloning proteins were detected by SEREX in sera from TIA patients. positive phage clones were marked by red arrows. **(B)** Positive clones obtained from above were rescreened to obtain monoclonality. **(C)** Positive clones from B were recloned to obtain monoclonality. **(D)** Antigenic proteins BRAT1 and WDR1 were succeeded in purification from precipitate fraction. **(E)** GST-BRAT1, GST-WDR1 and GST proteins were electrophoresed through SDS-polyacrylamide gels followed by staining with Coomassie Brilliant Blue (CBB), Western blotting using anti-GST (αGST), or sera of TIA patient [TIA#297]. The degradation products of GST-BRAT1 were marked by asterisks (*).

AS biomarkers were screened with SEREX (**Figures 1A–C**). Sera from the 19 patients in the TIA group were used for immunological screening. We identified certain clones and some of them were previously reported (32, 39). We focused on the antibody markers BRAT1 (accession No. NM_152743) and WDR1 (accession No. NM_017491). Full-length BRAT1 and WDR1 cDNAs were recombined into pGEX-4T-3 expression vectors. GST-labeled recombinant proteins were expressed in *E. coli* and purified by affinity chromatography with glutathione-Sepharose. Antigenic proteins were then purified from the precipitate fraction. The arrows in **Figure 1** indicate that the GST-fusion proteins could be detected in equal amounts in the total extracts, precipitates (Ppts), dialysates, and flow-through, purified, and concentrated samples but not in the supernatant (**Figure 1D**, middle).

Western blotting revealed antibodies against BRAT1 and WDR1 in the sera of patients with TIA. GST-BRAT1, GST-WDR1, and GST were recognized by anti-GST (αGST) antibody and detected as 58-kD, 59-kD, and 28-kD proteins, respectively (**Figure 1E**). The degradation products were marked with asterisks in **Figure 1E**. GST-BRAT1 and GST-WDR1 were recognized by serum IgG antibodies from a patient with TIA (TIA#297). There was no apparent reactivity against the serum IgG antibodies from the patients in the GST protein group (**Figure 1E**, right).

### Antibody Markers Against BRAT1 and WDR1 Are Predictors of TIA, aCI, and oCI Onset

**Figure 2:**
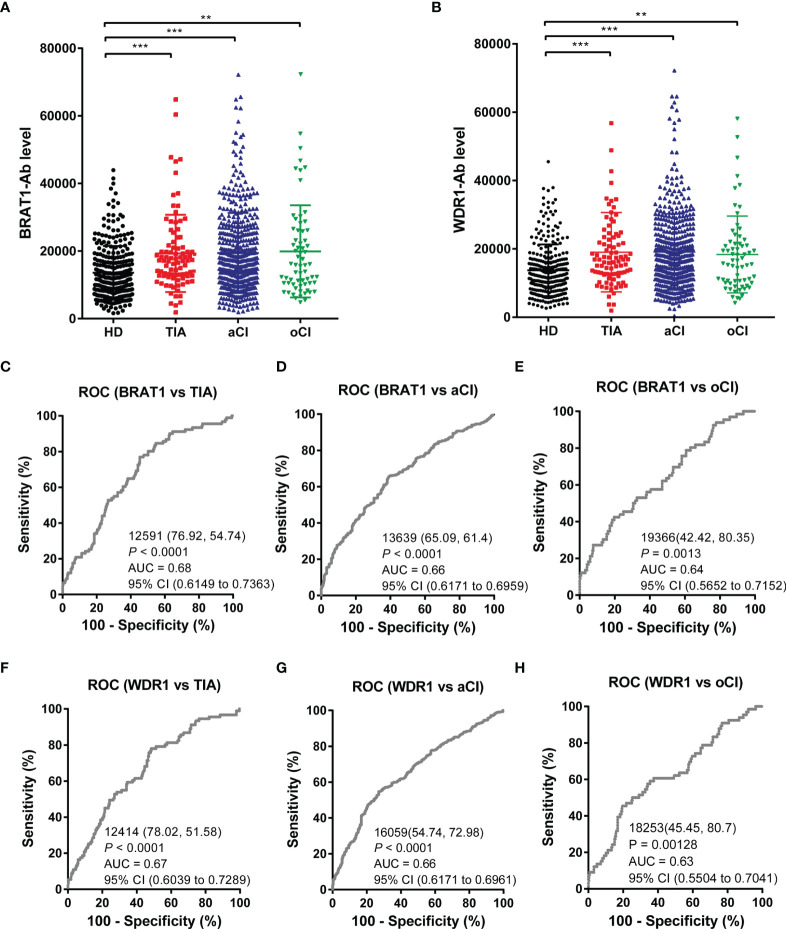
Comparison of serum anti-BRAT1 antibody (BRAT1-Abs) and anti-WDR1 antibody (WDR1-Abs) levels between healthy donors (HDs) and patients with TIA, aCI, or oCI. Serum antibody levels against BRAT1-GST **(A)** or WDR1-GST **(B)** were determined by AlphaLISA. The bars represent the median. *P* values were calculated by the Kruskal–Wallis test. **P < 0.01; ***P < 0.001. The serum numbers of HDs, TIA, aCI and oCI were 285, 91, 464 and 66, respectively. ROC analysis of BRAT1 and WDR1 for the prediction of TIA **(C, F)**, aCI **(D, G)** and oCI **(E, H)**. The numbers in the figures indicate the cutoff values for marker levels, and the numbers in parentheses indicate the sensitivity (left) and specificity (right).

We used AlphaLISA to measure serum BRAT1-Ab and WDR1-Ab in the HDs and patients with TIA, aCI, and oCI. Serum BRAT1-Abs and WDR1-Abs levels were significantly higher in the patients than the HDs (**Figures 2A, B**). **Table 1A** shows that there were 285, 92, 464, and 65 HDs and patients with TIA, aCI, and oCI, respectively. The distributions of men and women in these sample groups were 188/97, 55/37, 271/193, and 48/17, respectively. The average ages (± SD) in these treatment groups were 52.3 ± 11.7, 70.2 ± 11.6, 75.5 ± 11.5, and 73.3 ± 9.2 y, respectively. At the cutoff value, the positivity rates for BRAT1-Abs were 5.3, 14.1, 17.2, and 18.5% for the HDs and the patients with TIA, aCI, and oCI, respectively. The positivity rates for WDR1-Abs were 5.6, 14.1, 16.6, and 13.8% for the HDs and the patients with TIA, aCI, and oCI, respectively (**Table 1B**).

Table 1Comparison of serum BRAT1-, and WDR1-Ab levels between HDs and patients with TIA, aCI or oCI examined by AlphaLISA.

**Table:** 

A, Subject information on HDs and patients.				
Sample information	HD	TIA	aCI	oCI
Total sample number	285	92	464	65
Male/female	188/97	55/37	271/193	48/17
Age (average ± SD)	52.3 ± 11.7	70.2 ± 11.6	75.5 ± 11.5	73.3 ± 9.2

**Table:** B, Summary of serum BRAT1-, WDR1-Ab levels examined by AlphaLISA in HDs and patients.

Patient group	Type of value	BRAT1-Ab	WDR1-Ab
HD	Average	13,590	13,708
	SD	7,895	7,546
	Total No.	285	285
	Positive No.	15	16
	Positive rate	5.3%	5.6%
	Cutoff value	29,380	28,800
TIA	Average	19,224	18,990
	SD	11,378	11,545
	Total No.	92	92
	Positive No.	13	13
	Positive rate	14.1%	14.1%
	P value (TIA vs HD)	<0.001	<0.001
aCI	Average	19,418	19,101
	SD	11,856	11,154
	Total No.	464	464
	Positive No.	80	77
	Positive rate	17.2%	16.6%
	P value (aCI vs HD)	<0.001	<0.001
oCI	Average	20,005	18,386
	SD	13,718	11,327
	Total No.	65	65
	Positive No.	12	9
	Positive rate	18.5%	13.8%
	P value (oCI vs HD)	<0.01	<0.01

(A) The numbers of total samples, male/female participants, and ages [average ± SD] were showed. (B) The serum BRAT1-, WDR1-Ab levels were summarized respectively. Cut−off values were the average HD values plus two SDs, and positive samples for which the antibody levels exceeded the cutoff value were scored. P values were calculated using the Kruskal−Wallis test (Mann Whitney U with Bonferroni’s correction applied). Bold indicates P <0.05 and positive rates > 10%.

The abilities of BRAT1-Ab and WDR1-Ab to detect TIA, aCI, and oCI were evaluated by ROC analysis. In this manner, the efficacy of these markers at predicting TIA-related cardiovascular disease (CVD) was determined. The graphs in **Figures 2C**–**H** show the area under the curve (AUC), cutoff value, 95% CI, sensitivity, specificity, and *P-*value. The discriminant ability increases as the AUC value approaches unity (49). The BRAT1-Abs AUCs for TIA, aCI, and oCI were 0.68, 0.66, and 0.64, respectively (**Figures 2C**–**E**). Hence, BRAT1-Ab had good predictive efficacy for these diseases. The ROC analysis also disclosed that the WDR1-Abs AUCs were 0.67, 0.66, and 0.63 for TIA, aCI, and oCI, respectively (**Figures 2F**–**H**). Thus, BRAT1-Ab could effectively predict these conditions.

Spearman’s correlation analysis was performed to investigate the associations among the serum BRAT1-Ab and WDR1-Ab levels and the indices for the HDs and TIA, aCI, and oCI patients (**Table 2**). Both BRAT1-Ab and WDR1-Ab were correlated with age (r = 0.2165, *P* < 0.0001; r = 0.2263, *P*< 0.0001), HT (r = 0.1298, *P* < 0.0001; r = 0.1279, *P* < 0.0001), IMT (r) (r = 0.1986, *P* < 0.0001; r = 0.1928, *P* < 0.0001), ALP (r = 0.1348, *P* < 0.0001; r = 0.09273, *P* = 0.007), CRP (r = 0.175, *P* < 0.0001; r = 0.1547, *P* < 0.0001), IMT (l) (r = 0.2032, *P* < 0.0001; r = 0.1996, *P* < 0.0001), and IMT_max_ (r = 0.2068, *P* < 0.0001; r = 0.2059, *P* < 0.0001). BRAT1-Ab and WDR1-Ab were also correlated with height, weight, AST, LDH, WBC, RDW, BP, and smoking but not with alcohol consumption. In contrast, the antibody levels were negatively correlated with A/G, CHE, ALB, T-CHO, and RBC. The AS-associated parameter TG was negatively associated with the level of WDR1-Ab but not that of BRAT1-Ab. The BRAT1-Ab and WDR1-Ab levels were not significantly elevated in patients with DM (*P* = 0.213 and 0.079, respectively). There was no apparent positive correlation between the antibody levels and HbA1c (r = 0.01993, *P* = 0.6547; r = -0.00131, *P* = 0.9766).

**Table 2:** Correlation analysis between serum BRAT1-, and WDR1-Ab levels and the indices in HDs, TIA, aCI or oCI patients.

	BRAT1		WDR1	
	*r* value	*P* value	*r* value	*P* value
DE	-0.04979	0.0948	-0.06691	**0.0247**
Gender	0.02167	0.4629	0.008451	0.7747
Age	0.2165	**<0.0001**	0.2263	**<0.0001**
HT	0.1298	**<0.0001**	0.1279	**<0.0001**
CVD	0.06693	**0.0235**	0.06928	0.019
Lipidemia	-0.03442	0.2445	-0.05251	0.0757
Height (cm)	-0.1096	**0.0002**	-0.09368	**0.0016**
Weight (kg)	-0.08661	**0.0036**	-0.08623	**0.0037**
BMI	-0.01905	0.5234	-0.03352	0.2616
IMT (r)	0.1986	**<0.0001**	0.1928	**<0.0001**
IMT (l)	0.2032	**<0.0001**	0.1996	**<0.0001**
max IMT	0.2068	**<0.0001**	0.2059	**<0.0001**
A/G	-0.1621	**<0.0001**	-0.1579	**<0.0001**
AST	0.08959	0.0068	0.08846	**0.0076**
ALT	-0.005902	0.8589	-0.005971	0.8573
ALP	0.1348	**<0.0001**	0.09273	**0.007**
LDH	0.07935	**0.0184**	0.07601	**0.024**
tBil	-0.04618	0.1687	-0.01952	0.5608
CHE	-0.1308	**0.0005**	-0.1232	**0.0011**
gamma-GTP	0.02177	0.525	0.004055	0.9058
TP	-0.05725	0.0888	-0.05518	0.1009
ALB	-0.1462	**<0.0001**	-0.1451	<0.0001
BUN	0.02869	0.3878	0.04243	0.2015
Creatinine	0.01647	0.6209	0.02754	0.4083
eGFR	-0.02372	0.5007	-0.03562	0.3118
UA	0.06517	0.0931	0.04448	0.2521
AMY	-0.0194	0.6464	-0.03816	0.3665
T-CHO	-0.08879	**0.0125**	-0.1085	**0.0023**
HDL-c	-0.04374	0.2897	-0.03521	0.3941
TG	-0.0524	0.19	-0.08711	**0.0292**
Na	0.006592	0.8439	-0.01937	0.5627
K	-0.03535	0.2911	-0.05139	0.1247
Cl	0.00342	0.9186	-0.01637	0.6249
CRP	0.175	**<0.0001**	0.1547	**<0.0001**
WBC	0.09436	**0.0045**	0.09486	**0.0043**
RBC	-0.08239	**0.0132**	-0.07313	**0.0278**
HGB	-0.05905	0.0758	-0.04653	0.162
HCT	-0.05493	0.0987	-0.04934	0.1381
MCV	0.06051	0.0689	0.05869	0.0776
MCH	0.04304	0.1958	0.05706	0.0862
MCHC	-0.04678	0.1597	-0.01687	0.6123
RDW	0.1083	**0.0011**	0.09543	**0.0041**
PLT	-0.0327	0.3258	-0.05913	0.0754
MPV	-0.005877	0.8599	0.007393	0.8242
PCT	-0.03388	0.3086	-0.05629	0.0906
PDW	-0.02307	0.4882	-0.01689	0.6119
BS	0.0556	0.108	0.09597	**0.0055**
HbA1c	0.01993	0.6547	-0.00131	0.9766
BP	0.07989	**0.0444**	0.08173	**0.0397**
Smoking	0.1036	**0.0004**	0.08712	**0.0032**
Smoking period (year)	0.1197	**0.0014**	0.1075	**0.0042**
Alcohol	0.005809	0.8813	0.01715	0.6593
Alcohol Freq (time/w)	0.0062	0.8675	0.03706	0.3187

Subjects’ data were including age, height, weight, body mass index (BMI), Dialysis encephalopathy (DE), hypertension (HT), cardiovascular disease (CVD), maximum intima–media thickness (max IMT), intima–media thickness(right) (IMT (r)), intima–media thickness(left) (IMT (l)) albumin/globulin ratio (A/G), aspartate aminotransferase (AST), alanine amino transferase (ALT), alkaline phosphatase (ALP), albumin (ALB), lactate dehydrogenase (LDH), total bilirubin (tBil), cholinesterase (CHE), γ-glutamyl transpeptidase (γ-GTP), total protein (TP), albumin, blood urea nitrogen (BUN), estimated glomerular filtration rate (eGFR), uric acid (UA), amylase (AMY), total cholesterol (T-CHO), high-density lipoprotein cholesterol (HDL-C), triglyceride (TG), sodium (Na), potassium (K), chlorine (Cl), C-reactive protein (CRP), white blood cells (WBC), red blood cells (RBC), hemoglobin (HGB), hematocrit (HCT), mean corpuscular volume (MCV), mean corpuscular hemoglobin (MCH), MCH concentration (MCHC), red cell distribution width (RDW), platelets (PLT), mean platelet volume (MPV), procalcitonin (PCT), platelet distribution width (PDW), blood sugar (BS), and glycated hemoglobin (HbA1c), blood pressure (BP). Correlation coefficients (r values) and P values obtained by Spearman’s correlation analysis. Bold indicates P < 0.05.

The foregoing results suggest associations among the BRAT1-Ab and WDR1-Ab levels and CVD including TIA, aCI and oCI. Moreover, BRAT1-Ab and WDR1-Ab are potential molecular markers for TIA, aCI, and oCI onset.

### Serum BRAT1 and WDR1 Antibody Levels Are Elevated in Patients With Atherosclerotic AMI

**Figure 3:**
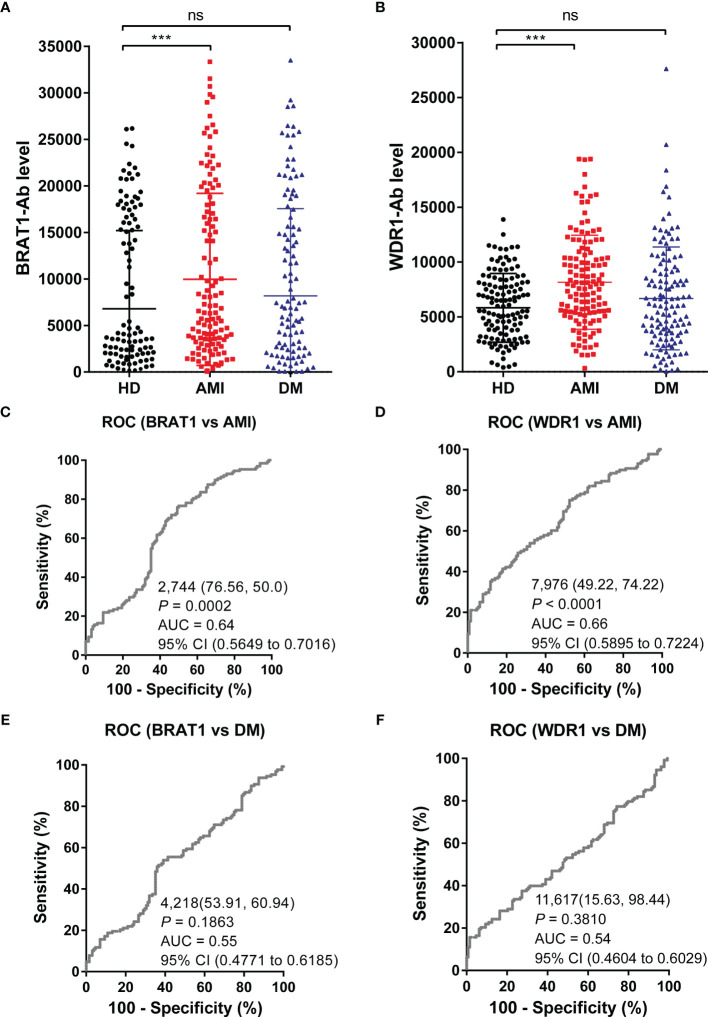
Comparison of serum BRAT1-Abs and WDR1-Abs levels between HDs and patients with AMI or DM. Serum antibody levels against BRAT1-GST **(A)** or WDR1-GST **(B)** were detected by AlphaLISA. The bars represent the median. *P* values were calculated by the Kruskal–Wallis test. ***P < 0.001. ns, not significant. The serum number of HDs, AMI, and DM was 128. ROC analysis of BRAT1 and WDR1 for the prediction of AMI **(C, D)** and DM **(E, F)**. The numbers in the figures indicate the cutoff values for marker levels, and the numbers in parentheses indicate the sensitivity (left) and specificity (right).

The serum antibody levels in the HDs and the AMI and DM patients were detected with AlphaLISA to verify the ability of BRAT1-Ab and WDR1-Ab to detect AS-associated diseases. Serum BRAT1-Ab and WDR1-Ab levels were significantly higher in AMI patients than HDs but not DM patients (**Figures 3A, B**). At the cutoff value, the BRAT1-Ab positivity rates were 3.1%, 10.2%, and 8.6% for the HDs, AMI, and DM, respectively. For WDR1-Ab, the positivity rates were 1.6%, 16.4% and 14.1% for the HDs, AMI, and DM, respectively (**Table 3**). Hence, serum BRAT1-Ab and WDR1-Ab more effectively predict CVD than DM.

**Table 3:** Comparison of serum BRAT1-, and WDR1-Ab levels between HDs and patients with AMI or DM tested by AlphaLISA.

Patient group	Type of value	BRAT1-Ab	WDR1-Ab
HD	Average	6795.4	5834.0
	SD	8403.8	3131.0
	Total No.	128	128
	Positive No.	4	2
	Positive rate	3.1%	1.6%
	Cutoff value	23603.0	12095.9
AMI	Average	9958.0	8158.0
	SD	9249.2	4288.8
	Total No.	128	128
	Positive No.	13	21
	Positive (%)	**10.2%**	**16.4%**
	P value (AMI vs HD)	**<0.01**	**<0.001**
DM	Average	8184.0	6713.7
	SD	9378.1	4691.3
	Total No.	128	128
	Positive No.	11	18
	Positive (%)	8.6%	14.1%
	P value (DM vs HD)	0.213	0.079

P values were calculated using the Kruskal−Wallis test (Mann Whitney U with Bonferroni’s correction applied). P < 0.05 and positive rate > 10% are marked in bold font.

The ROC analysis parameters including AUC, 95% CI, cutoff value, sensitivity, specificity, and *P* value are shown in **Figures 3C**–**F**. The AUCs of BRAT1-Ab and WDR1-Ab for AMI were 0.64 (95% CI = 0.5649–0.7013) and 0.66 (95% CI = 0.5895–0.7224), respectively. The AUCs of BRAT1-Ab and WDR1-Ab for DM did not significantly increase to > 0.6 and were only 0.55 (95% CI = 0.4771–0.6185) and 0.54 (95% CI = 0.4604–0.6029), respectively.

The foregoing results indicate that serum BRAT1-Ab and WDR1-Ab effectively predict the onset of atherosclerosis-related diseases.

### BRAT1 Expression Levels Were Significantly Elevated in GI Cancer Tissues

**Figure 4:**
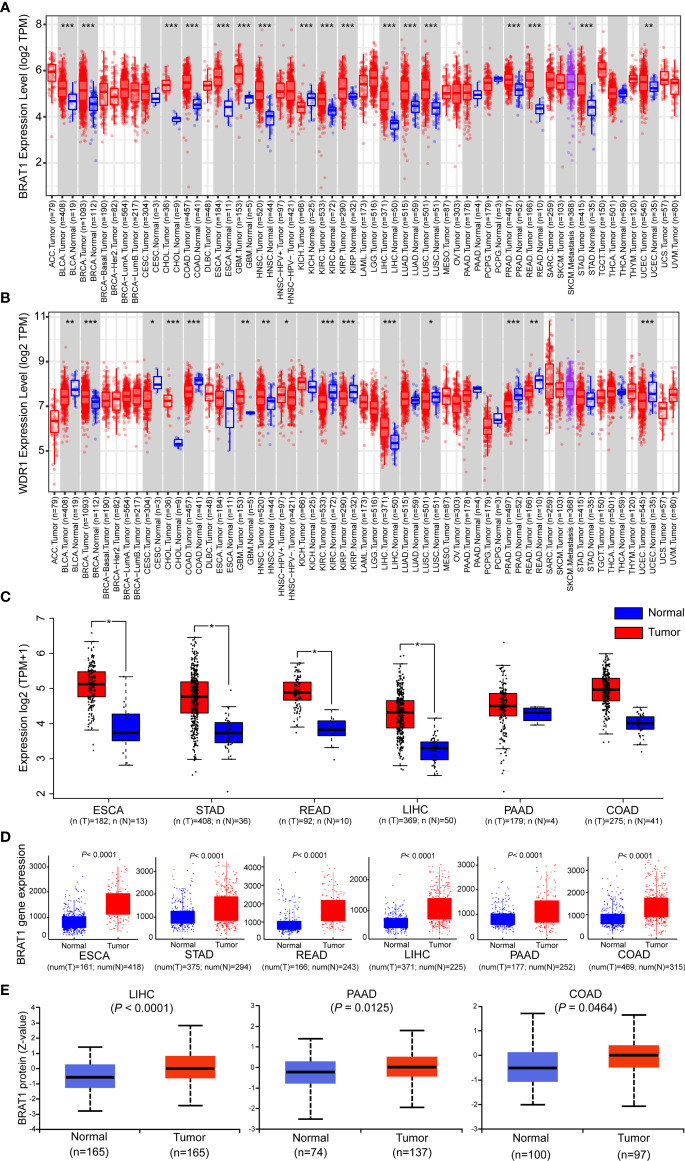
Comparison of BRAT1 levels between normal and gastrointestinal cancer tissues. **(A, B)** The expression levels of human BRAT1 and WDR1 in different cancer types were obtained from TCGA data in TIMER. **(C)** For the main type of gastrointestinal cancer, including ESCA, STAD, READ, LIHC, PAAD and COAD in the TCGA project, the normal tissues of the TCGA normal data were as controls. The box plot data were obtained from GEPIA web-based tool. **(D)** Plots of BRAT1 expression in normal and gastrointestinal cancer tissues of based on gene chip data of TNMplot. **(E)** BRAT1 proteomic expression profile in gastrointestinal cancers, LIHC, PAAD and COAD from CPTAC samples. Standard deviations from the median across samples for the given cancer types were represented by Z values. n represents the number of samples. *P < 0.05, **P < 0.01, ***P < 0.001.

AS-associated diseases and cancers are major global causes of mortality and morbidity. They share common modifiable pathogenesis risk factors. Thus, the prophylactic strategies used against atherosclerotic vascular disease may also be efficacious against cancers (6, 50). We used the TIMER2.0 database to analyze the mRNA expression levels across all TCGA tumors and identify the differences in BRAT1 and WDR1 expression between tumors and adjacent normal tissues. **Figure 4A** shows that relative to adjacent normal, healthy tissues, BRAT1 was significantly upregulated in BLCA (bladder urothelial carcinoma), BRCA (breast invasive carcinoma), CHOL (cholangiocarcinoma), COAD (colon adenocarcinoma), ESCA (esophageal carcinoma), GBM (glioblastoma multiforme), HNSC (head and neck squamous cell carcinoma), KICH (kidney chromophobe), KIRC (kidney renal clear cell carcinoma), KIRP (kidney renal papillary cell carcinoma), LIHC (liver hepatocellular carcinoma), LUAD (lung adenocarcinoma), LUSC (lung squamous cell), PRAD (prostate adenocarcinoma), READ (rectum adenocarcinoma), STAD (stomach adenocarcinoma), and UCEC (uterine corpus endometrial carcinoma). BRAT1 was significantly upregulated in all five types of GI cancer (COAD, ESCA, LIHC, READ, and STAD). Contrastingly, WDR1 was significantly upregulated only in LIHC (**Figure 4B**). Therefore, the expression of BRAT1 but not WDR1 was positively correlated with GI cancers. As the statistical strategies and sample sizes differed between the TIMER2.0 and GEPIA databases, the latter was used to confirm BRAT1 upregulation in ESCA, STAD, READ, LIHC, PAAD, and COAD and their adjacent normal tissues. Matched TCGA normal data were used as controls. (**Figure 4C**). RNA-seq data generated by the TNMplot online tool showed that BRAT1 was upregulated in six different GI cancers compared with normal tissues (**Figure 4D**). We also measured BRAT1 protein expression in normal and LIHC, PAAD, and COAD tissues using Clinical Proteomic Tumor Analysis data (**Figure 4E**).

The preceding results acquired from multiple online databases indicated high BRAT1 expression in GI cancers and suggested that BRAT1 might play a crucial role in serum detection of these diseases.

### Serum BRAT1-Ab Levels Are Potential Predictors of GI Cancers

**Figure 5:**
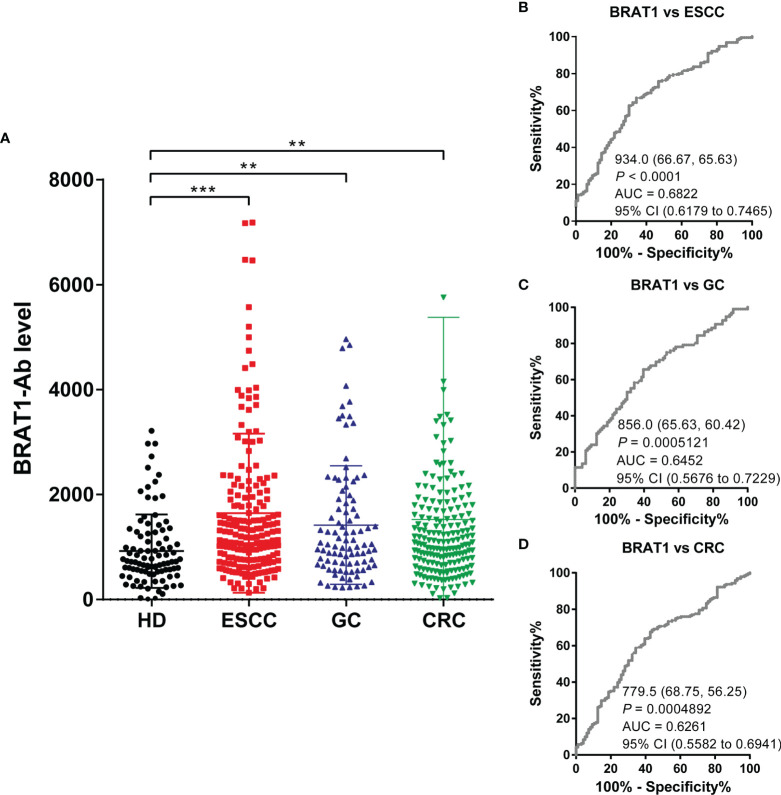
Comparison of serum BRAT1-Abs levels between HDs and patients with gastrointestinal cancers. **(A)** Serum antibody levels against BRAT1-GST were determined by AlphaLISA. The bars represent the median. The Kruskal–Wallis test was used for calculating *P* values. The serum number of HDs, ESCC, GC and CRC were 96, 192, 96 and 192, respectively. sensitivity and specificity of BRAT1 between ESCC **(B)**, GC **(C)**, CRC **(D)** were evaluated by ROC analysis. Numbers in the figure represent cutoff level, specificity and sensitivity. **P < 0.01, ***P < 0.001.

AlphaLISA analysis was performed on HDs and patients with ESCC, GC, and CRC to establish whether serum BRAT1-Ab is a novel serum biomarker of these GI cancers (**Figure 5A** and **Table 4**). Serum BRAT1-Abs levels were significantly higher for patients with ESCC, GC, and CRC than the HDs. Serum WDR1-Abs levels were markedly elevated in patients with ESCC and GC but not in those with CRC (**Supplementary Figure 1A**; **Supplementary Table 1**). At a cutoff value of the mean HD value plus 2 SD, the BRAT1-Abs positivity rates for the HDs and patients with ESCC, GC, and CRC were 6.3%, 17.9%, 15.6%, and 11.5%, respectively (**Table 4**). The WDR1-Abs positivity rates for the HDs and patients with ESCC, GC, and CRC were 4.3%, 27.7%, 17.4%, and 16.0%, respectively (**Supplementary Table 1**).

**Table 4:** Comparison of serum BRAT1-Ab levels between HDs and patients with ESCC, GC or CRC examined by AlphaLISA.

Patient group	Type of value	BRAT1-Ab
HD	Average	923
	SD	700
	Cutoff value	2,324
	Total no.	96
	Positive no.	6
	Positive rate	6.3%
ESCC	Average	1,635
	SD	1,512
	Total no.	192
	Positive no.	34
	Positive rate	**17.7%**
	P value (ESCC vs HD)	**<0.001**
GC	Average	1,417
	SD	1,130
	Total no.	96
	Positive no.	15
	Positive rate	**15.6%**
	P value (GC vs HD)	**<0.001**
CRC	Average	1,526
	SD	3,903
	Total no.	192
	Positive no.	22
	Positive rate	**11.5%**
	P value (CRC vs HD)	**<0.05**

P values were calculated using the Kruskal−Wallis test (Mann Whitney U with Bonferroni’s correction applied). Bold indicates P < 0.05 and positive rates > 10%.

We then used ROC analysis to assess the abilities of these markers to detect GI cancers. **Figures 5B**–**D** show that the AUCs of BRAT1-Abs for ESCC, GC, and CRC were 0.68 (95% CI = 0.6203–0.7489), 0.64 (95% CI = 0.5676–0.7229), and 0.62 (95% CI = 0.5560–0.6924), respectively. The AUCs of WDR1-Abs for ESCC, GC, and CRC were 0.68 (95% CI = 0.6233–0.7479), 0.68 (95% CI = 0.6131–0.7651), and 0.59 (95% CI = 0.5306–0.6646), respectively (**Supplementary Figures 1B**–**D**). The cutoff value, sensitivity, specificity, and *P* value are shown under the curves. Significant increases > 0.6 were only observed for the AUCs of BRAT1-Ab vs. ESCC, GC, and CRC. In contrast, the AUC of WDR1-Ab vs. CRC was < 0.6.

The foregoing findings revealed that relative to WDR1-Ab, BRAT1-Ab is a superior predictor of GI cancers and the AS-associated biomarker BRAT1-Ab is a potential predictor of the onset of ESCC, GC, and CRC.

### Serum BRAT1−Ab Levels Are Positively Correlated with Overall Survival

**Figure 6:**
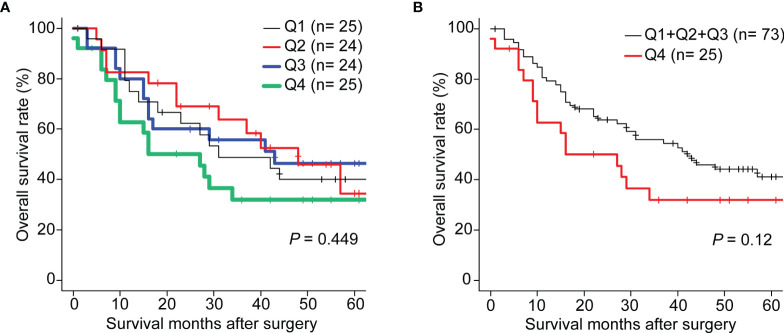
Comparison of overall survivals of the patients with ESCC according to BRAT1-Abs levels. Kaplan-Meier plots are shown. The number of patients was shown in parentheses. **(A)** The BRAT1-Abs levels were classified into every one-fourth quartiles according to antigen level (Q1, Q2, Q3 and Q4). **(B)** The BRAT1-Abs levels were classified into two groups (Q1+Q1+Q3 vs. Q4). The *p* value at 60 months after surgery was 0.12. Log-Rank test was performed to compare the difference between two groups.

The AUC values were highest for ESCC (**Figure 6**). Thus, 98 surgical ESCC cases were analyzed and used to validate the correlations between BRAT1-Ab and overall survival. We divided the BRAT1−Ab levels for ESCC into quartiles Q1 (n = 25), Q2 (n = 24), Q3 (n = 24), and Q4 (n = 25). There were no statistically significant differences in OS among groups (**Figure 6A**) according to a log-rank test (*P* = 0.12) (**Table 5**, left panel). However, the Q4 group presented with poor ESCC prognosis at 5–60 wks post-surgery (**Figure 6B**). The foregoing results show that the highest serum BRAT1-Ab levels (Q4) were associated with poor ESCC prognosis.

**Table 5:** Univariate and multivariate analysis of risk factors for overall survival in the patients with ESCC.

	Univariate analysis	Multivariate analysis		
	*P* value^a^	Hazard ratio	95% CI^b^	*P* value^c^
Gender	**0.04**	0.71	0.31-1.62	0.42
Male/Female				
Age	0.58			
>65/≤65				
Location	0.30			
Upper/Lower				
Tumor depth	**<0.01**	2.45	1.07-5.60	**0.03**
T 1/T2-4				
Tumor depth	**<0.01**			
T1-2/T3-4				
Lymph node metastasis	**<0.01**	2.03	1.01-4.07	0.05
N-/N+				
SCC-Ag (ng/mL)	**0.02**	1.17	0.65-2.11	0.60
>1.5/≤1.5				
p53-Abs (U/mL)	0.06			
>1.30/≤1.30				
WBC (/μL)	0.81			
>8000/≤8000				
Neutrophil (%)	0.16			
>70/≤70				
Lymphocyte (%)	0.42			
>35/≤35				
Hemoglobin (g/dL)	0.08			
≤12/>12				
Platelet	0.17			
≤150000/>150000				
CRP (mg/dL)	**<0.01**	1.80	0.99-3.27	0.05
>0.3/≤0.3				
Albumin (g/dL)	0.22			
≤3.5/>3.5				
BRAT1-Ab	0.12	1.18	0.59-2.36	0.64
Q4/Q1Q2Q3				

SCC-Ag, squamous cell carcinoma antigen. ^a^Log-rank test; ^b^Adjusted 95% confidence interval; ^c^Cox proportional hazard model. Bold indicates a P < 0.05.

### High Serum BRAT1-Ab Levels Are Correlated With Clinicopathological Factors

We applied various statistical methods to investigate the correlations among the serum BRAT1-Ab levels and the clinicopathological factors. In a Cox proportional hazards regression analysis, tumor depth, SCC-Ag, and BRAT1-Ab were the explanatory variables and gender, age, location, lymph node metastasis, p53-Abs level, WBC, neutrophils, lymphocytes, hemoglobin, platelets, CRP, and albumin were tested (**Table 5**). A multivariate survival analysis only disclosed statistically significant correlations between the serum BRAT1-Ab levels and lymph node metastasis (*P* = 0.05), CRP level (*P* = 0.05), and tumor depth (*P* = 0.03). Hence, these parameters were designated as independent prognostic factors (**Table 5**, right panel).

Fisher’s exact probability test showed that the BRAT1-Ab levels were not associated with the aforementioned factors (**Table 6**, left panel). A logistic regression analysis demonstrated that platelet count was significantly correlated with high serum BRAT1-Ab levels (**Table 6**, right panel). A Mann-Whitney *U* test returned similar results for the associations between the foregoing clinicopathological factors and the median BRAT1−Ab levels in ESCC. Only platelet count and high serum BRAT1-Ab level were significantly correlated (**Table 7**).

**Table 6:** Comparison of serum BRAT1-Abs levels quartiles according to clinicopathological characters of the patients with ESCC.

Variables		Fisher’s exact probability test^a^			Logistic regression analysis^b^		
		BRAT1	BRAT1	*P* value	odds ratio	95% CI	*P* value
		Q1+Q2+Q3	Q4				
Gender	Male	56	20	1			
	Female	17	5				
Age	>65	42	15	1			
	≤65	31	10				
Location^c^	Upper	10	5	0.53			
	Lower	60	20				
Tumor depth^c^	T1	25	5	0.21	1.52	0.43-5.41	0.52
	T2-T4	44	19				
Lymph node metastasis	N0	29	13	0.48			
	N1	41	12				
WBC(/μL)^c^	>8000	10	2	0.72			
	≤8000	60	22				
Neutrophil (%)	>70	15	4	0.77			
	≤70	55	20				
Lymphocyte (%)	>35	12	5	0.76			
	≤35	58	19				
Hemoglobin (g/dL)	>12	46	16	1			
	≤12	24	8				
Platelet	>150000	67	20	0.07	6.03	1.05-34.7	**0.04**
	≤150000	3	4				
CRP (mg/dL)^c^	>0.3	19	10	0.13	2.89	0.95-8.82	0.06
	≤0.3	50	12				
Albumin (g/dL)	>3.5	55	16	0.28	1.68	0.51-5.60	0.40
	≤3.5	15	8				
SCC-Ag (ng/mL)^c^	>1.5	23	11	0.32			
	≤1.5	45	12				
p53-Abs (U/mL)^c^	>1.30	16	2	0.14	0.32	0.06-1.66	0.18
	≤1.30	52	22				

a Fisher’s exact probability test;

b Logistic regression analysis;

c Loss value. Bold indicates P < 0.05.

**Table 7:** Comparison of serum BRAT1-Ab levels median according to clinicopathological characters of the patients with ESCC.

Variables		Number of patients	Median (min-max)	*P* value^a^
Gender	Male	76	1275(128-11718)	0.33
	Female	22	998(210-7173)	
Age	>65	57	1132(128-6475)	0.48
	≤65	41	1308(216-11718)	
Tumor depth	T1	30	1083(216-7173)	0.11
	T2 T3 T4	63	1353(199-11718)	
Tumor depth	T1 T2	38	1102(199-7173)	0.25
	T3 T4	55	1353(210-11718)	
Lymph node status	Negative	42	1373(199-11718)	0.25
	Positive	53	1132(210-7185)	
Location	Upper	15	1295(216-4997)	0.44
	Lower	80	1159(199-11718)	
WBC(/μL)	>8000	12	948(210-7185)	0.70
	≤8000	82	1223(199-11718)	
Neutrophil (%)	>70	19	804(210-11718)	0.30
	≤70	75	1254(199-7185)	
Lymphocyte (%)	>35	17	1185(491-6475)	0.71
	≤35	77	1192(199-11718)	
Hemoglobin (g/dL)	>12	62	1275(199-7185)	0.72
	≤12	32	1084(210-11718)	
Platelet	>150000	87	1111(199-11718)	**<0.01**
	≤150000	7	2371(1353-4997)	
CRP (mg/dL)^b^	>0.3	29	1185(304-11718)	0.72
	≤0.3	62	1162(199-7173)	
Albumin (g/dL)	>3.5	71	1218(304-7185)	0.97
	≤3.5	23	1082(199-11718)	
SCC-Ag (ng/mL)^b^	Negative	57	1074(199-7173)	0.06
	Positive	34	1361(298-7185)	
p53-Abs (U/mL)^b^	Negative	74	1331(199-7185)	0.28
	Positive	18	1018(216-11718)	

a Mann-Whitney U Test;

b Loss value. Bold indicates P < 0.05.

## Discussion

As cancers and AS-associated disease pathogenesis share common modifiable risk factors, predictive strategies of atherosclerotic vascular disease could also conceivably be used to detect cancers (50–52). We applied western blot on the sera of patients with TIA, used SEREX screening, and identified the antigens BRAT1 and WDR1 (**Figure 1**). Subsequent analyses established elevated serum BRAT1-Abs and WDR1-Abs in patients with TIA, aCI, oCI, and AMI but not in those with DM compared with HDs (**Tables 1**, **3**; **Figures 2A, B**, **3A, B**). ROC and Spearman’s correlation analyses showed that BRAT1-Abs and WDR1-Abs could detect atherosclerotic vascular diseases (**Figures 2** and **3**). For this reason, serum BRAT1-Abs and WDR1-Abs are potential AS biomarkers. We used online databases and AlphaLISA detection to compare protein (**Figure 4**) and serum antibody (**Figure 5A**) expression levels and found BRAT1 and BRAT1-Abs upregulation in patients with GI cancers. Significant increases > 0.6 were always observed for the AUCs of BRAT1-Ab vs. the GI cancers ESCC, GC, and CRC (**Figures 5B–D**; **Supplementary Figures 1B–D**). Thus, BRAT1-Ab more effectively predicts GI cancers than WDR1-Ab. A log-rank test revealed no significant differences in OS among the Q1+Q2+Q3 and Q4 groups (*P* = 0.12) (**Table 5**, left panel). Nevertheless, the highest serum BRAT1-Ab levels (Q4 group) were associated with poor prognosis at 5–60 wks after ESCC surgery (**Figure 6**). We verified the foregoing conclusion by comparing serum BRAT1-Ab levels among ESCC patients according to their clinicopathological characteristics. Multiple statistical strategies demonstrated and confirmed a correlation between the BRAT1-Ab level and the platelet count (**Tables 5**–**7**). To the best of our knowledge, this study is the first to determine by AlphaLISA detection that serum BRAT1-Ab and WDR1-Ab are elevated in patients with atherosclerotic diseases and can be used as predictors for them. Furthermore, the AS-related biomarker BRAT1-Ab could serve as a predictive risk marker for GI cancers.

As obesity and insulin resistance have become epidemic, there is growing evidence that hypertriglyceridemia is a risk factor for AS (53). Spearman’s correlation analysis was performed to examine the associations among the serum BRAT1-Ab and WDR1-Ab levels and the indices for HDs and TIA, aCI, and oCI patients (**Table 2**). The levels of both BRAT1-Ab and WDR1-Ab were correlated with most atherosclerotic parameters. In contrast, TG was negatively associated with the WDR1-Ab level. Therefore, the WDR1-Ab levels may not directly reflect AS. Rather, they might indirectly reflect the lesions caused by AS. Moreover, BRAT1-Ab may be a better predictor of atherosclerotic diseases than WDR1-Ab.

Platelets regulate thrombosis and hemostasis. Nevertheless, a few studies suggested that crosstalk between tumor cells and platelets facilitates cancer progression and metastasis (54, 55). Tumorigenesis is accompanied by thrombosis and thromboembolism. Hence, platelet-tumor aggregates regulate platelet function by and altering their cancer-mediated and releasing platelet granules (56, 57). Platelets also activate endothelial cells, recruit immunocytes, and facilitate tumor cell spread (58). Platelets enhance tumor growth, invasion, and metastasis by promoting proliferation, antiapoptosis, pro-angiogenic signals, and the invasive tumor cell epithelial-mesenchymal transition (EMT) phenotype (59–62). Activated platelets also secrete transforming growth factor β (TGF-β) into the tumor microenvironment (TME), suppress tumor immunity, and favor cancer cell evasion of the host immune system (63). As there is complex, bidirectional communication between platelets and cancer cells, platelet count elevation is an important cancer marker in primary care. Even a marginal increase in platelet count is correlated with a clinically relevant increase in cancer risk (64). In GI cancers, the upregulation of platelet-dependent signaling and tyrosine phosphatase facilitates changes in aggressive cancer phenotypes (65). Platelet count elevation indicates poor OS and is a predictive biomarker of digestive malignant tumors (66). Platelet activation is correlated with locally advanced ESCC and predicts long-term OS especially in nodal-positive patients (67, 68). Here, logistic regression analysis (**Table 6**) and Mann-Whitney *U* tests (**Table 7**) revealed that the BRAT1-Ab levels were significantly correlated with the platelet counts in ESCC. There is no direct evidence for any correlation between the BRAT1 levels and the platelet counts. Nevertheless, the foregoing results demonstrate that BRAT1 plays a crucial role in GI cancers such as ESCC.

A previous study reported a progressive linear relationship between increased platelet count and EC stage. While patients in the more advanced stages presented with thrombocytosis, those in the earlier stages did not (81.81% in stage III and 100% in stage IV). Patients with thrombocytosis also had pathological lymph node metastases (69). Platelet counts were significantly elevated in patients with deep and large tumors. High platelet counts were associated with tumor progression and low survival rates in patients with EC (70, 71). Contrastingly, platelet counts were significantly reduced in patients undergoing neoadjuvant chemoradiotherapy (CRT). Hence, platelet counts could help estimate the response of patients with EC to CRT (72). Patients with EC who are undergoing concurrent chemoradiotherapy usually present with thrombocytopenia (73). Contrastingly, platelets are key growth factor sources and could promote tumor angiogenesis and invasion. Hence, EC development may rely on platelet-mediated growth factor signal transduction (72, 74). Therefore, EC treatment might benefit patients by reducing platelet counts (74). In conclusion, platelet count is associated with tumor progression in EC, helps predict therapeutic outcomes, and could be utilized in multimodal treatment regimens to provide precise personalized cancer treatment (75).

BRAT1 is a binding partner of BRCA1 and participates in mitochondrial homeostasis, DNA damage response, and cell growth apoptosis (76). In BRAT1 knockout (KO) cancer cell lines, the ROS levels were increased and mitochondrial membrane potential and ATP production decreased. Therefore, BRAT1 plays key roles in cancer cell mitochondrial function (77). Moreover, BRAT1 protein is oncogenic in several different cancers (78). *In vitro* and *in vivo* cell proliferation and tumorigenicity were significantly reduced in BRAT1 KO cancer cell lines (77). Curcusone D promoted DNA repair and inhibited cancer cell migration by downregulating BRAT1 (78). Mitochondrial function plays important roles in AS diseases and cancers. In fact, mitochondrial dysfunction has been observed in both conditions (79–81). No correlation between BRAT1 and AS has been reported. However, BRAT1 may play vital roles in AS and cancer by influencing mitochondrial function.

Oxidative stress-induced DNA damage is associated with both AS and cancer pathogenesis and promotes their progression (51, 82, 83). The stress-induced DNA double-strand breaks upregulate endogenous BRAT1, which, in turn, increases cell survival by regulating ATM phosphorylation (84). BRAT1 overexpression may stimulate an autoimmune response and induce AS- and GI cancer-associated BRAT1 autoantibodies. This mechanism might explain why serum BRAT1-Abs levels are elevated in patients with AS and GI cancers (**Figures 2**, **5**; **Table 2**).

The immune system participates in pathophysiological processes and promotes increase in the levels of certain autoantibodies (85). Possible mechanisms causing elevated autoantibody levels in tumors include host immune responses to tumor-associated antigens (TAAs), pathological immune dysregulation, and antigenic stimulation induced by malignant cell destruction (86). AS is associated with chronic inflammation, has characteristics resembling those of autoimmune diseases, and is always accompanied by the formation of various antigens such as oxidized low-density lipoproteins (LDL) and heat shock proteins (HSP) related to autoimmunity (17). The levels of autoantibodies against LDL and HSP increase in the sera of patients with atherosclerotic diseases (87).

Autoantibodies are highly stable (half-life ≤ 30 d) and durable in serum samples (88). The immune system amplifies certain autoantibodies in response to a single autoantigen. Repeated exposure of immunocytes to even small amounts of antigen induces abundant antibody production and may raise serum autoantibody levels. Antibody biomarkers increase detectable signals of their corresponding antigens, are more sensitive than antigen markers, and are, therefore, potential diagnostic markers (85, 89). Tumor-associated autoantibodies are produced early during tumorigenesis, can be measured before clinical symptoms appear (90), are early indicators of abnormal cellular processes during tumorigenesis, and are associated with malignant transformations (91). The early stages of AS are sometimes accompanied by low levels of tissue destruction, protein leakage from disrupted cells, and elevated autoantibody expression (92). AS autoantibodies are the driving factors of inflammation, risk factors of AS-related diseases, and protective (anti-AS) factors. Hence, they are closely related to AS occurrence and development (93) and could be used in early disease screening and to monitor disease progression (94).

Here, BRAT1-Ab was screened by liquid biopsy SEREX analysis and identified in patients with TIA which is a prodromal AS symptom. BRAT1-Ab was consistently upregulated in AS-related diseases and GI cancers. Serum BRAT1-Ab is a potential diagnostic biomarker of TIA, aCI, oCI, AMI, ESCC, GC, and CRC. BRAT1-Ab upregulation may predict the early onset of AS and GI cancers. Furthermore, early BRAT1-Ab detection could help prevent disease onset and support the application of liquid biopsy in AS and GI cancers.

## Data Availability Statement

The original contributions presented in the study are included in the article/**Supplementary Material**. Further inquiries can be directed to the corresponding authors.

## Ethics Statement

This study involving human participants was reviewed and approved by the Local Ethical Review Board of the Graduate School of Medicine, Chiba University (No. 2012–438, No. 2014–44, No. 2016–86, No. 2017–251, No. 2018–320, No. 2020–1129) and Jinan University (JNUKY-2021-045), and Ethics Committee of Toho University, Graduate School of Medicine (nos. A19033), as well as the cooperating Chiba Prefectural Sawara Hospital, Chiba Rosai Hospital, Chiba Aoba Municipal Hospital, and Port Square Kashiwado Clinic (Japan). Recombinant DNA studies were performed with the official permission of the Graduate School of Medicine (Chiba University, Japan) and Jinan University (China), and conducted in conformity with the rules of the Japanese and Chinese government. The patients/participants provided their written informed consent to participate in this study.

## Author Contributions

All authors contributed to the article and approved the submitted version. LH, JYL, HW, and SS contributed to the project development and research, manuscript writing. QZ, JSL, and KS edited the manuscript. TH, HW, KS, HS, and MI collected the serum sample. TH, HW, LH, HS, and MI contributed toward the statistical analysis of this work. LH, JYL interpreted the data.

## Funding

This study was funded by National Science Foundation, China (Grant No. 82071340); Natural Science Foundation of Guangdong Province, China (Grant No. 2018A0303131003); Medical Science and Technology Research Fund of Guangdong Province, China (Grant No. A2019550).

## Conflict of Interest

The authors declare that the research was conducted in the absence of any commercial or financial relationships that could be construed as a potential conflict of interest.

## Publisher’s Note

All claims expressed in this article are solely those of the authors and do not necessarily represent those of their affiliated organizations, or those of the publisher, the editors and the reviewers. Any product that may be evaluated in this article, or claim that may be made by its manufacturer, is not guaranteed or endorsed by the publisher.
