# Supplementary material for: Serum Anti-BRAT1 is a Common Molecular Biomarker for Gastrointestinal Cancers and Atherosclerosis
Source: Front Oncol. 2022 May 17;12:870086. doi: 10.3389/fonc.2022.870086 (PMC9152111; doi:10.3389/fonc.2022.870086)
Supplement: Supplementary file 1 [file DataSheet_1.docx]

Supplementary Material

# Supplementary Figures and Tables

## Supplementary Figure


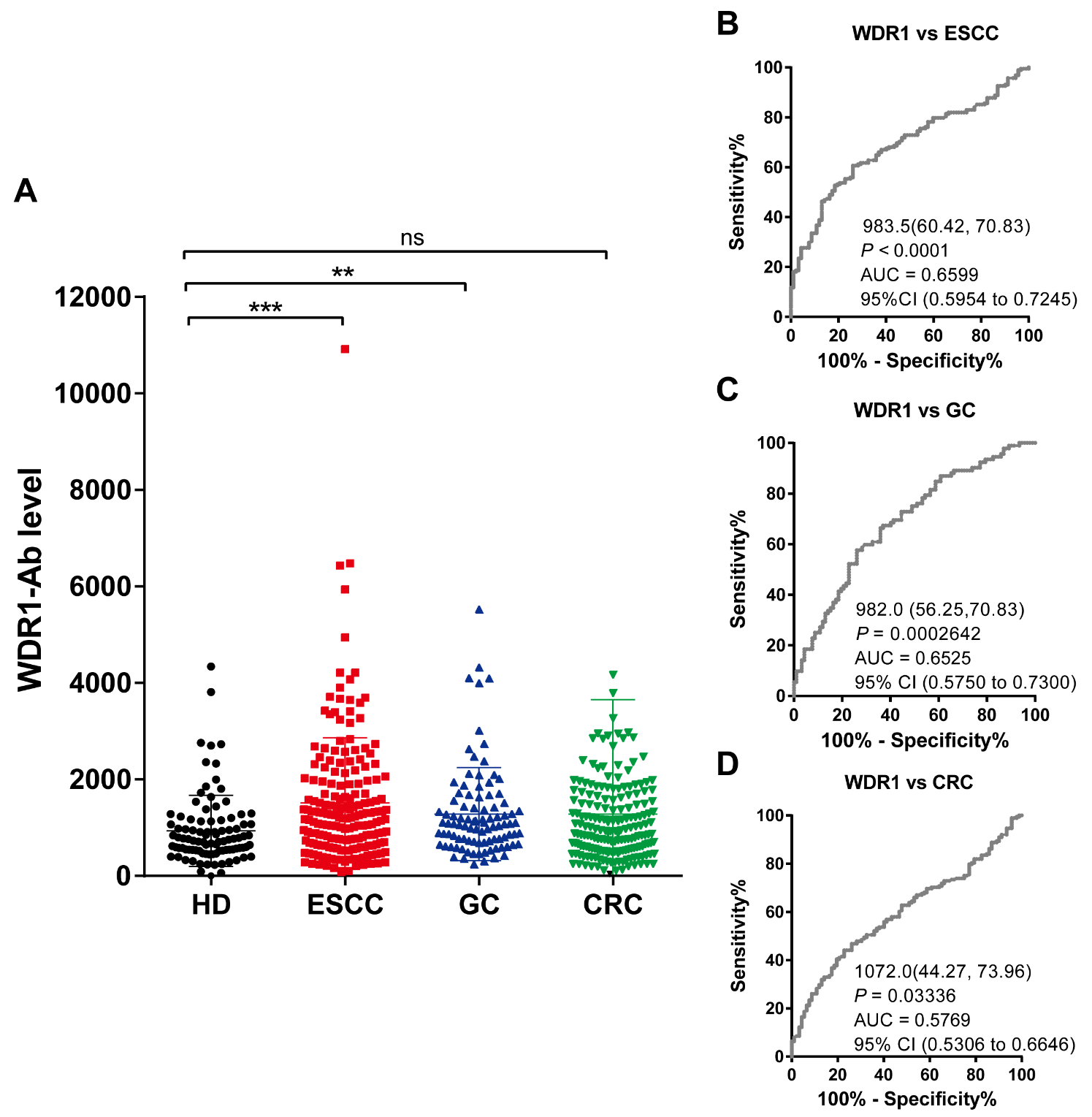


**Supplementary Figure 1.** **Comparison of serum anti-WDR1 antibody (WDR1-Abs) levels between healthy donors (HDs) and patients with ESCC, GC, and CRC.** **(A)** AlphaLISA-determined serum antibody levels against WDR1-GST after subtraction of the levels against those of control GST are shown. The bars represent the median. *P* values were calculated by the Kruskal–Wallis test. ***P* < 0.01, ****P* < 0.001. The serum number of HDs, ESCC, GC and CRC were 96, 192, 96 and 192, respectively. The other information including total (male/female) numbers, average values, SDs, cutoff values, positive numbers, positive rates (%), and *P* values is summarized and shown in Table 4. ROC analysis was performed to evaluate sensitivity and specificity of WDR1 between esophageal cancer **(B)**, gastric cancer **(C)**, colorectal cancer **(D)**. Numbers in the figure represent cutoff level, specificity and sensitivity, the areas under the curve (AUC), 95% confidence intervals (CI) and *P* value.

## Supplementary Table

**Supplementary Table 1 Comparison of serum WDR1-Ab levels between HDs and patients with ESCC, GC or CRC examined by AlphaLISA**

| Patient group | Type of value | WDR1-Ab |
| --- | --- | --- |
| **HD** | Average | 935 |
|  | SD | 739 |
|  | Cutoff value | 2,412 |
|  | Total no. | 96 |
|  | Positive no. | 5 |
|  | Positive rate | 5.2% |
| **ESCC** | Average | 1,516 |
|  | SD | 1,348 |
|  | Total no. | 192 |
|  | Positive no. | 33 |
|  | Positive rate | **17.2%** |
|  | P value (ESCC vs HD) | **<0.001** |
| **GC** | Average | 1,281 |
|  | SD | 967 |
|  | Total no. | 96 |
|  | Positive no. | 9 |
|  | Positive rate | **9.4%** |
|  | P value (GC vs HD) | **<0.01** |
| **CRC** | Average | 1,281 |
|  | SD | 2,374 |
|  | Total no. | 192 |
|  | Positive no. | 14 |
|  | Positive rate | 7.3% |
|  | P value (CRC vs HD) | 0.16 |

The antigens used were purified WDR1-GST protein. The shown numbers are as described in Table 1; *P* values were calculated using the Kruskal‑Wallis test (Mann Whitney U with Bonferroni's correction applied). *P* values lower than 0.05 and positive rates higher than 10% are marked in bold.
